# Expression of protein kinase C gamma promotes cell migration in colon cancer

**DOI:** 10.18632/oncotarget.18916

**Published:** 2017-07-01

**Authors:** Catríona M. Dowling, Sheri L. Hayes, James J. Phelan, Mary Clare Cathcart, Stephen P. Finn, Brian Mehigan, Paul McCormick, John C. Coffey, Jacintha O'sullivan, Patrick A. Kiely

**Affiliations:** ^1^ Graduate Entry Medical School, University of Limerick, Limerick, Ireland; ^2^ Health Research Institute University of Limerick, Limerick, Ireland; ^3^ Trinity Translational Medicine Institute, Department of Surgery, Trinity College Dublin, St. James's Hospital, Dublin, Ireland; ^4^ Department of Histopathology, St James's Hospital, Trinity College Dublin, Ireland; ^5^ GEMS, St. James Hospital, Dublin, Ireland

**Keywords:** protein kinase C gamma, tumor promoter, colon cancer

## Abstract

Despite extensive efforts, Protein Kinase Cs (PKCs) have proven to be an intractable target in cancer therapies. Traditionally it was accepted that PKCs act as tumour promoters, however new research suggests that PKCs may play an important role in the suppression of cancer. A challenge in targeting PKCs is the limited data available in patient samples. One of the PKC isozymes, PKC gamma, is thought to be present only in the brain and has been largely neglected in the context of cancer. Analysis of gene expression levels of PKC gamma in patient matched normal and colon cancer tissue samples revealed an up-regulation of the gene in the cancer tissue of 54% of the patients examined. Mechanistically we demonstrate that a reduction in the levels of PKC gamma in the colon cancer cells inhibits cell migration and foci formation. Further to this, we observe an increase in cell adhesion and proliferation following the reduction of PKC gamma levels in the cell. Thus, PKC gamma plays a key role in colon cancer; making it an important isozyme that needs to be reconsidered in the context of cancer therapies.

## INTRODUCTION

The Protein Kinase C (PKC) family have been studied extensively as a group of proteins that promote cancer. This follows the initial discovery that PKCs are a receptor for the tumour-promoting phorbal esters [1–3]. This led to the development of several target therapies against PKCs in cancer [4–6], however recent evidence has emerged suggesting that PKCs can play a tumour suppressor role in cancer [7–9].

PKCs are a multigene family of serine/threonine kinases which together are expressed in a diverse range of tissues and have several biological functions. The 9 PKC isozymes mediate several biological processes including cell adhesion, the cell-cycle and the regulation of apoptosis [10, 11]. All members of the PKC family share common basic structures; a flexible hinge segment linking a cell membrane targeting N-terminal regulatory moiety to a C-terminal catalytic domain [12, 13]. The regulatory moiety contains two discrete membrane targeting modules and a pseudosubstrate segment that maintains the enzyme in an inactive conformation [13, 14]. The maturation of PKCs into this conformation is dependent on sequential phosphorylation steps at three highly conserved sites termed the activation loop, the turn motif, and the hydrophobic motif [15–17]. Following these phosphorylation events, the protein is activated by specific secondary messengers that bind to the regulatory domain to mediate the release of the pseudosubstrate segment from the active site [14, 18]. The 9 isozymes are classified according to their secondary messenger requirements; the conventional isozymes (cPKCs: α, βI, βII, and γ) depend on diacylglycerol (DAG) and Ca^2+^ for their activation, the novel isozymes (nPKCs: δ, ε, η, and θ) depend on DAG, but not Ca^2+^, and the atypical isozymes (aPKCs: ζ and λ/ι) do not require either DAG or Ca^2+^ [10, 19, 20].

Although PKCs have been studied intensively, their precise role in cancer remains elusive [20]. This is largely due to the difficulty in deciphering the specific role of individual isozymes. Immunohistochemical and biochemical studies indicate that altered expression of the PKC isozymes is variable and depends on the cancer type [4, 21, 22]. We have shown recently that PKC Beta II is downregulated in colon cancer which promotes IGF-I-mediated cell survival [7]. In other studies, it has been shown that PKCζ overexpression in colon cancer cell lines decreases tumour formation in nude mice while loss of PKCζ is also associated with decreased tumourigenicity [23, 24]. Further to this, PKCα can both induce and supress colon cancer cell proliferation [25, 26]. The expression of PKCβ in breast, gastric and colon cancer has been subject to much debate and there are many studies presenting arguments for both up and down regulation of the isozyme in cancer cells and cancer tissue [5, 7, 27–31]. Collectively, these studies led to the development of several target therapies against cancer [4–6]. However, PKC inhibitors have proved unsuccessful as anti-cancer agents in clinical trials [4, 32] and recent evidence has come to the fore suggesting that PKCs can play a tumour suppressor role in cancer [7–9].

What is now clear is that the PKC isozymes have distinct functions. Clearly defining the expression pattern of individual PKC isozymes in cancer is essential in deciphering whether PKCs exhibit a tumour suppressor and/or a tumour promotor role. Traditionally, it was believed that PKC gamma is expressed only in the brain [33, 34] and consequentially it received very little attention in the context of cancer. However, in recent times some evidence has emerged suggesting that PKC gamma is also present in colon tissue [35] and colonic cells [36]. Moreover, further novel research indicates that PKC gamma influences the migratory capacity of many cell types [37–39]. Taken together, this challenges the view that PKC gamma is expressed only in the brain and suggests that a recalibration of our understanding of PKC gamma is important as we revisit the role of PKCs in cancer.

In our study, we examined the expression of PKC gamma in colon cancer *in vivo* and *in vitro* to fully elucidate if the gene was present or absent in colon tissue and to understand whether its function was enhanced or reduced in this cancer. Keeping in mind that PKCs are differentially expressed across and within different tissues, our approach was to reduce any variability that may occur between patients by examining the expression of the PKC gamma coding gene in patients matched normal and colon cancer tissue. Interestingly, not only was the PKC gamma gene present in the tissue but it was also significantly up-regulated in the colon cancer tissue compared to the normal tissue in over 50% of patients. To examine the functional implications of this, using siRNA knockdown for PKC gamma, we demonstrate that PKC gamma influences the transformed phenotype and more specifically, increases the migratory capacity of colon cancer cells. All this data combined, suggests that PKC gamma plays a role in promoting colon cancer.

## RESULTS

### The gene coding PKC gamma is up-regulated in colon cancer

**Figure 1:**
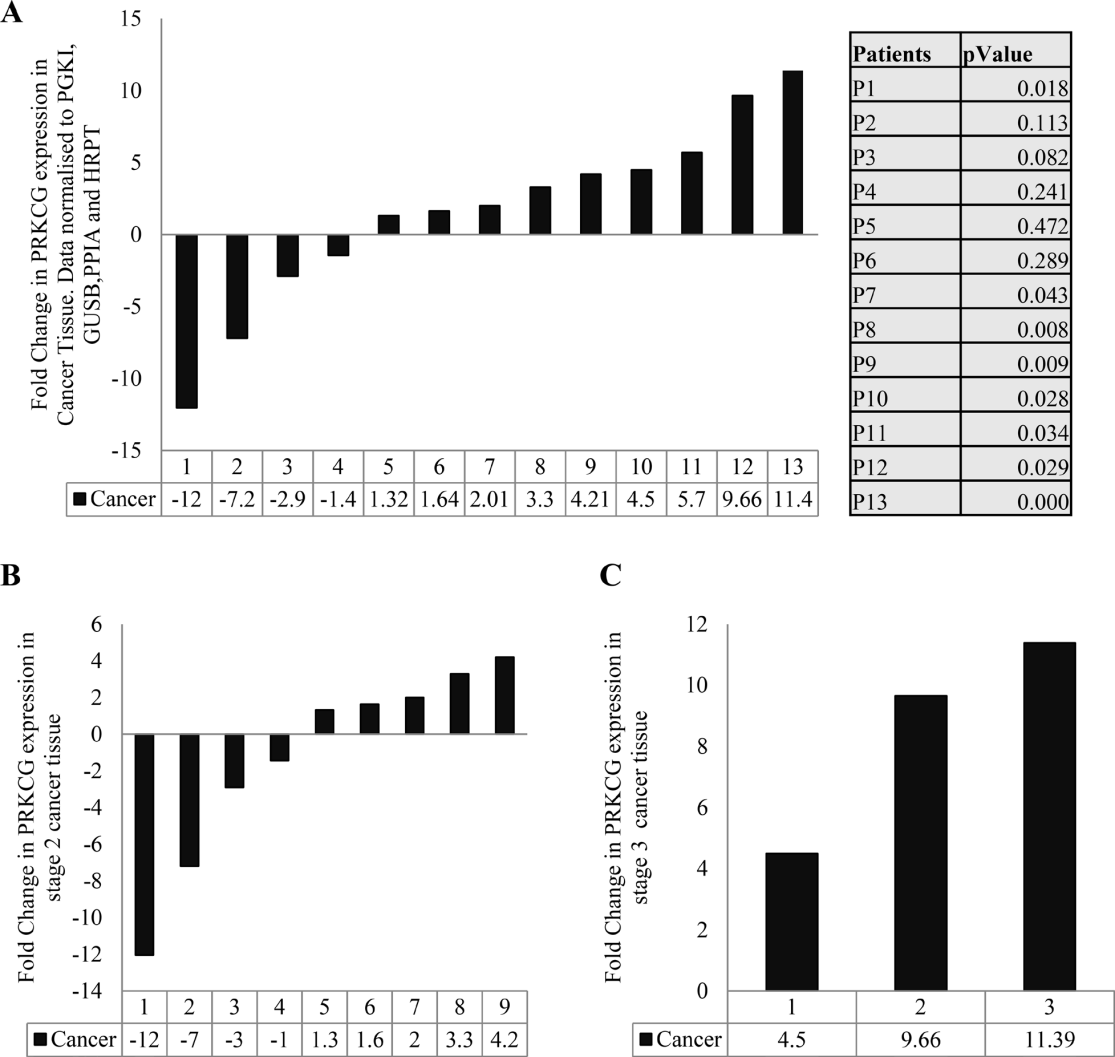
Gene expression of PKC genes in colon cancer Tissue samples measuring approximately 0.5cm in diameter were collected from 23 patients undergoing surgery in University Hospital Limerick. Normal tissue from the 23 patients was also collected approximately 10 cm away from the cancer tissue. RNA was extracted from the tissue, cDNA was synthesised and real time PCR was carried out. All data was normalized using the housekeeping genes PRGK1, GUSB, PPIA and HRPT1. (**A**) Fold change of PRKCG (PKC Gamma coding gene) in cancer tissue of each patient. Results were obtained by comparing mRNA level of PRKCG in individual's normal tissue compared to levels in that individual's cancer tissue (Statistical difference based on Pair Wise Fixed Reallocation Randomisation Test^©^ as per REST^©^ software). (**B**) Fold change of PRKCG in stage 2 cancer tissue. (**C**) Fold change of PRKCG in stage 3 cancer tissue (No statistical difference found between stages as determined by Mann Whitney *U* test).

We have previously shown differential expression of the PKC isoforms in colon cancer [7]. To investigate the expression of the PKC gamma coding gene, we compared the expression of the gene in patients’ cancer tissue to patients’ matched normal distant tissue. We detected the gene coding PKC gamma in 13 out of a total of 23 patient samples and found that the presence or absence of the gene was not influenced by disease stage (Table 1). Taking the 13 patient samples in which the gene was expressed, we next established an individual fold change for each patient. The results show that 54% of the patients demonstrate a significant up-regulation of the PKC gamma coding gene (Figure 1A). This up-regulation was not influenced by the progression of the disease as no significant difference was found between the percentages of patients with an up-regulation in stage 2 or stage 3 of the disease (Figure 1B).

**Table 1:** Characteristics of the cohort of colorectal cancer (CRC) patients used to analysis the mRNA levels of PKC gamma

Patient Number	Gender	Age	Stage	PRKCG*
1	M	55	1	+
2	F	45	2	+
3	M	68	2	+
4	M	85	2	+
5	M	79	2	+
6	F	83	2	+
7	M	82	2	+
8	M	82	2	+
9	F	53	2	+
10	M	65	2	+
11	M	77	2	–
12	F	59	2	–
13	M	80	2	–
14	F	63	2	–
15	M	81	2	–
16	M	70	3	+
17	M	56	3	+
18	F	69	3	+
19	F	66	3	–
20	M	59	3	–
21	M	68	3	–
22	F	69	3	–
23	M	59	3	–

^*^+ and − refer to absolute presence or absence of PRKCG.

### Loss of PKC gamma influences the malignant phenotype in colon cancer cells

Our data indicates that there is an up-regulation of the gene coding PKC gamma in over 50% of the colon cancer patients in our study. Considering this, we questioned if the loss of PKC Gamma would influence the transformed phenotype *in vitro*.

**Figure 2:**
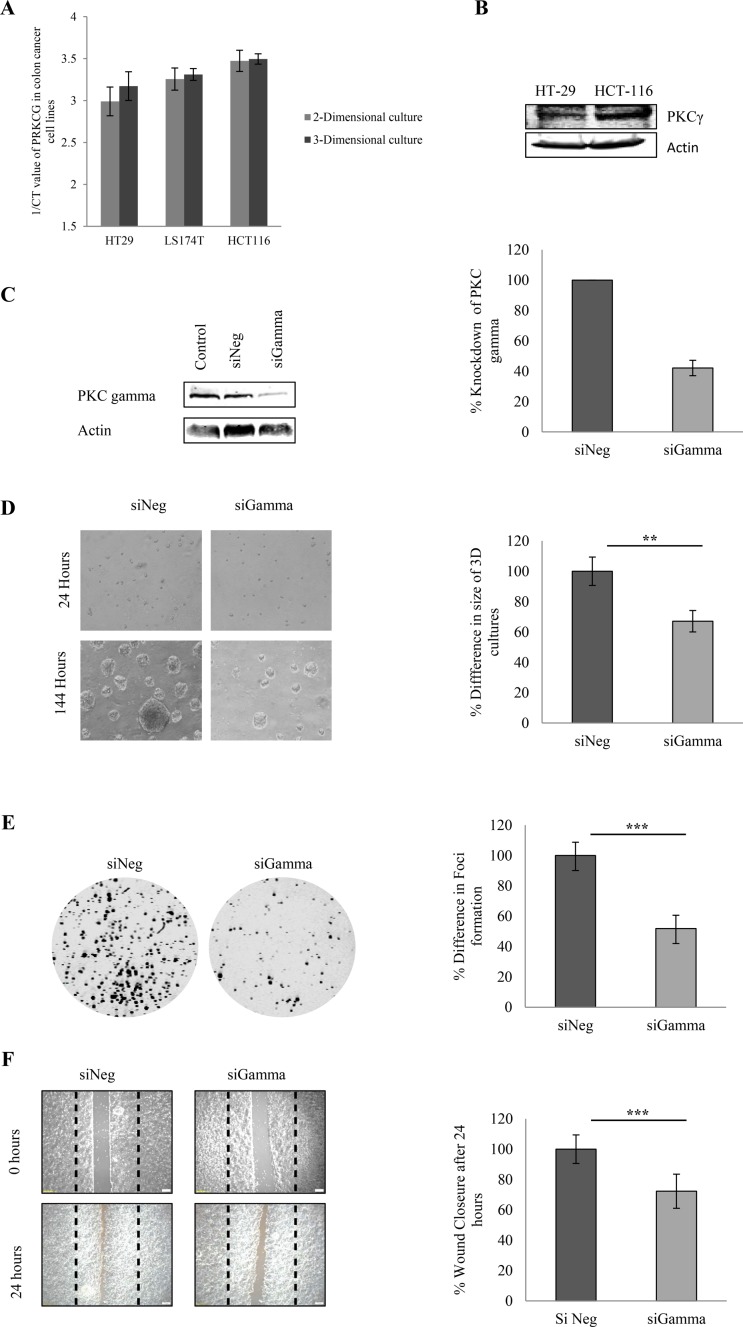
Effect of PKC gamma knockdown on colon cancer cells The effect of knockdown of PKC Gamma in colon cancer cells was examined using HCT116 cells transfected with an siRNA against PKC gamma. (**A**) The inverse of the Ct value obtained for the mRNA levels of PKC Gamma in HT29, LS174T and HCT116 cell lines. (**B**) Representative western blot demonstrating the expression of PKC Gamma in HT29 and HCT116 cell lines. (**C**) Representative western blot showing the expression percentage knockdown of PKC Gamma in HCT116 cells. (**D**) Images demonstrating the difference in the size of cells growing in a 3D matrix over 144 hours. Bar graph represents the percentage difference in the size of 3D cultures at 144 hours (Statistical difference based on Mann Whitney *U* test, **p < 0.01). (**E**) Representative images demonstrating the difference in the cells ability to form colonies when PKC gamma is reduced (Statistical difference based on Mann Whitney *U* test, ***p < 0.001). (**F**) Representative image of cells that were scored with a wound and allowed to migrate over 24 hours. Bar graph represents the percentage wound closer after 24 hours (Statistical difference based on Mann Whitney *U* test, ***p < 0.001).

Firstly, in order to select the cells which expressed the highest levels of PKC gamma we examined the levels of the gene coding for PKC gamma in three different colon cancer cell lines maintained in either 2-Dimensional or 3-Dimensional growth conditions (Figure 2A). To further validate, this we also examined the protein expression of PKC Gamma in HT29 and HCT116 cell lines (Figure 2B). Together, the results demonstrate that HCT116 show the highest expression of PKC gamma at both a gene and protein level. Considering this, we reduced levels of PKC gamma in HCT116 cells using siRNA oligonucleotides (Figure 2C) and performed different functional cell based assays.

We developed spheroids of the HCT116 cells and using these 3-dimensional cultures, we observed that loss of PKC gamma in these cells resulted in a significant reduction in the size of the spheroids (Figure 2D, Supplementary Figure 1). Following this, we examined if the loss of PKC gamma would influence the cells ability to form colonies. Transfected HCT116 cells were seeded at very low densities and their capacity to develop colonies was monitored over a period of 10 days. Interestingly, cells demonstrated a significant reduction in colony size and number when PKC gamma levels were reduced in the cell (Figure 2E). We next tested if PKC gamma loss would influence the migratory capacity of HCT116 cells. To conduct this, we utilized ibidi^®^ inserts and carried out a wound migration assay. Transfected HCT116 cells were seeded around the insert at 100% confluency, 24 hours later the insert was removed and cells were monitored as they migrated into the space created by the insert. Here, we observed a significant reduction in the ability of cells to migrate into the wound when PKC gamma levels were reduced (Figure 2F). Taken together, this data strongly supports the hypothesis that PKC gamma promotes a transformed phenotype.

### Loss of PKC gamma enhances the proliferation and adherence of colon cancer cells

**Figure 3:**
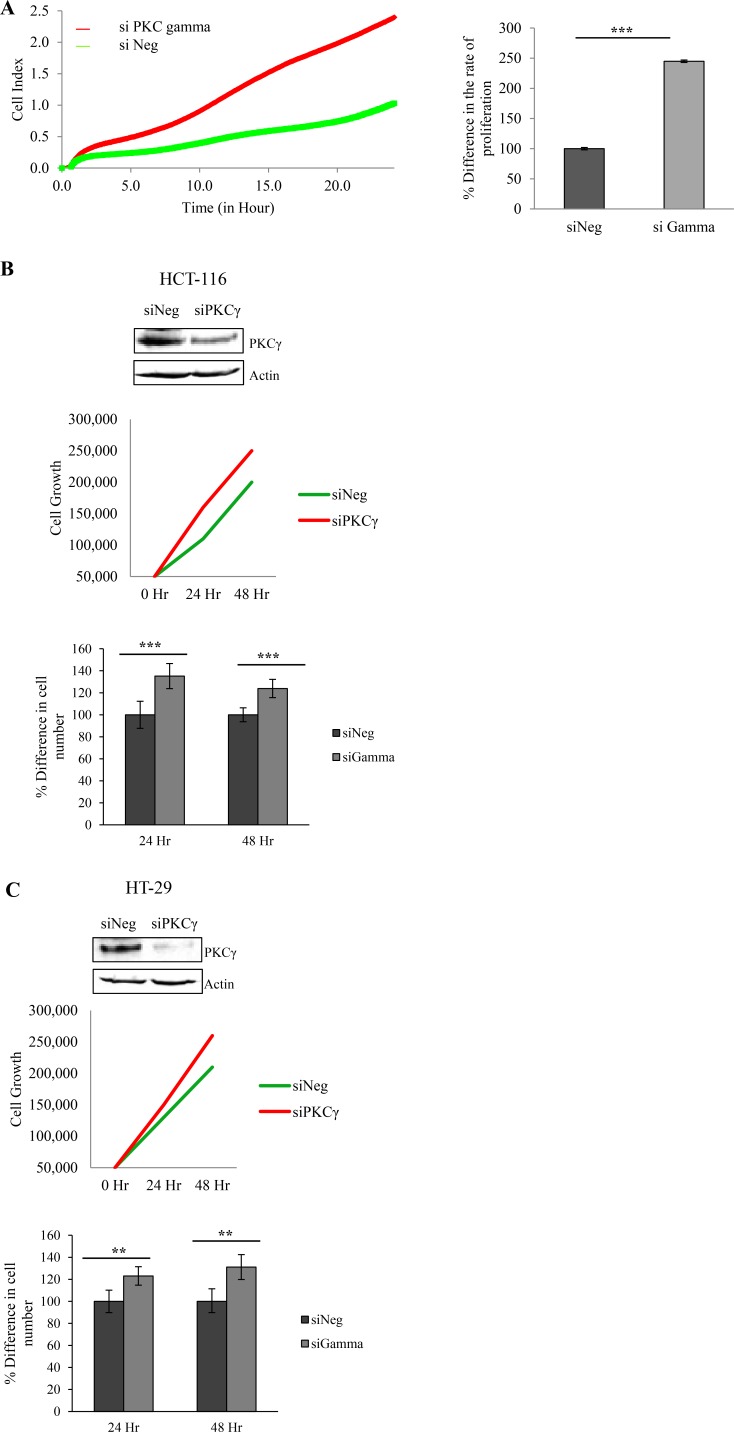
Effect of PKC gamma knockdown on proliferation of colon cancer cells The effect of knockdown of PKC Gamma on proliferation in colon cancer cells was examined using the real-time cell analysis xCELLigence system and traditional cell growth assays. (**A**) Representative graph of HCT116 cells proliferating over a period of 24 hours analysed in real time on the xCELLigence system. Bar graph represents the rate of proliferation as determined by averaging the cell index over 24 hours (Statistical difference based on Mann Whitney *U* test, ***p < 0.001). (**B**) Graph indicating cell growth at 24 hr and 48 hr in HCT116 cells as determined by the traditional cell growth assay. Bar graph represents the percentage difference in cell number at 24 hr and 48 hr (Statistical difference based on Mann Whitney *U* test, ***p < 0.001). (**C**) Graph indicating cell growth at 24 hr and 48 hr in HT29 cells as determined by the traditional cell growth assay. Bar graph represents the percentage difference in cell number at 24 hr and 48 hr (Statistical difference based on Mann Whitney *U* test, **p < 0.01).

**Figure 4:**
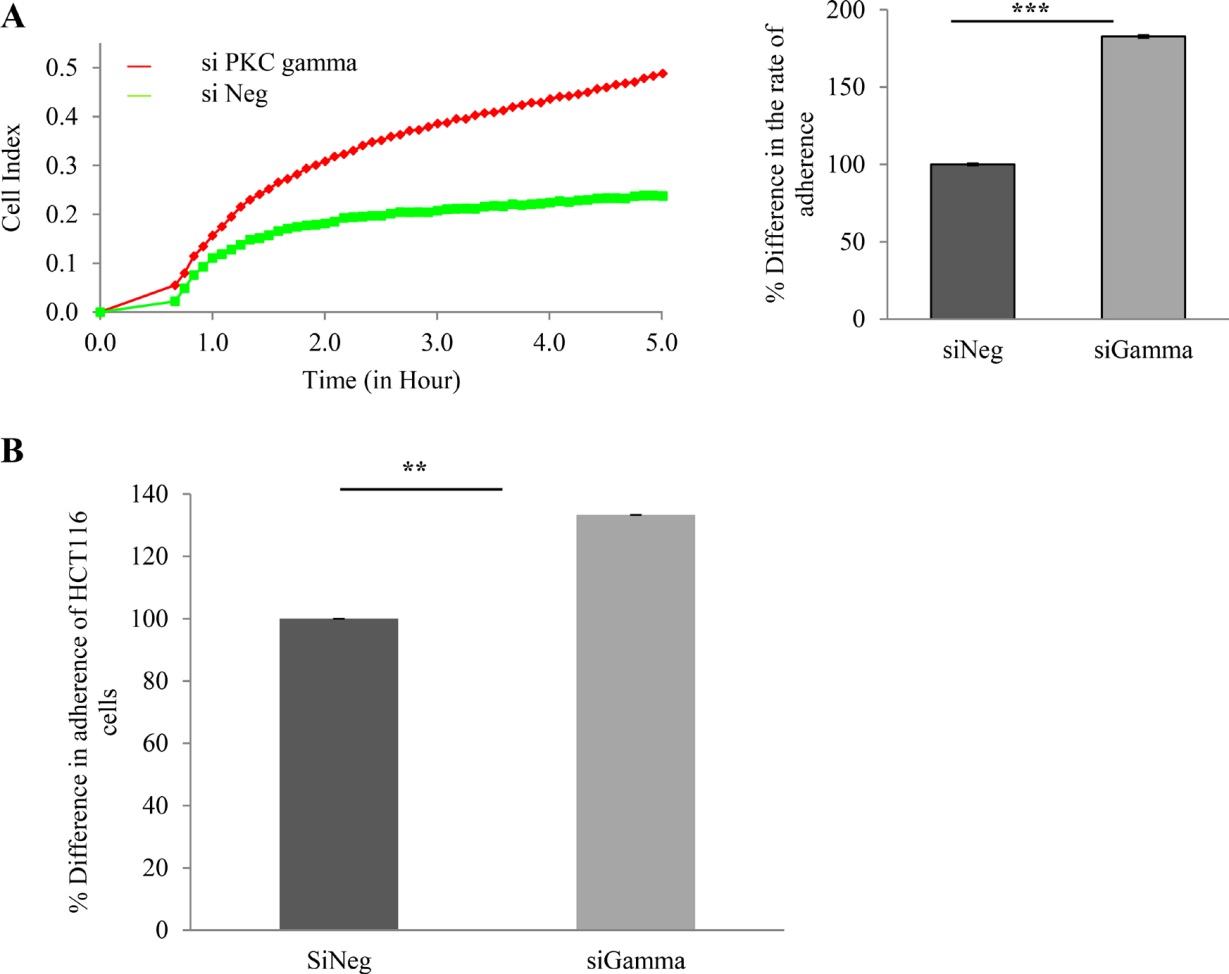
Effect of PKC gamma knockdown on adherence of colon cancer cells The effect of knockdown of PKC Gamma on adherence in colon cancer cells was examined using the real-time cell analysis xCELLigence system and traditional cell adherence assays. (**A**) Representative graph of HCT116 cells adhering over a period of 5 hours analysed in real time on the xCELLigence system. Bar graph represents the rate of adhesion determined by averaging the cell index over 5 hours (Statistical difference based on Mann Whitney *U* test, ***p < 0.001). (**B**) Bar graph showing the difference in cell adherence for crystal violet stained HCT116 cells, absorbance was determined at 595 nm after 3 hours (Statistical difference based on Mann Whitney *U* test, **p < 0.01).

Having shown that loss of PKC gamma inhibits cell migration and foci formation, we next wanted to investigate the consequences of this loss on cell proliferation and adhesion. To do this, cellular levels of PKC gamma were reduced using a siRNA oligonucleotide. Cells were plated in wells of an E-plate and their behaviour was monitored using RTCA as previously described [40]. Results indicate that HCT116 cells proliferate significantly faster when PKC gamma is reduced in the cells (Figure 3A). To further validate this we conducted a traditional cell count assay and again we observed an increase in cell number when PKC gamma is reduced in both HCT116 cells (Figure 3B) and HT29 cells (Figure 3C). To investigate the effects on cell adhesion, data was extracted from the platform over the first 5 hours of cell monitoring. Results show that HCT116 cells in which PKC gamma levels have been reduced adhere almost twice as fast as cells treated with the scrambled siRNA oligonucleotide (Figure 4A). To confirm this, we used a traditional assay to show that loss of PKC gamma in HCT116 cells results in a 40% increase in cell adhesion (Figure 4B). This data suggests that PKC gamma plays an important role in regulating the proliferation and adhesion of colon cancer cells.

## DISCUSSION

Using tissue samples we show that the gene coding for PKC gamma is up-regulated in 54% of patient's colon cancer tissue when compared to patient matched normal tissue. Further to this, suppression of PKC gamma reduces the migratory capacity of colon cancer cells. This study challenges traditional views that PKC gamma is present only in the brain and elucidates to a role for PKC gamma in driving the transformed phenotype in colon cancer.

Our comprehensive gene expression analysis identified the presence of the gene coding PKC gamma in 57% of patient's normal colon tissue and colon cancer tissue. This finding is intriguing as PKC gamma was traditionally thought to be only expressed in the brain [33, 34] and has also been used as a negative control for the colon [41]. Moreover, when comparing the expression of the PKC gamma coding gene in individual patients matched normal and colon cancer tissue we revealed an up-regulation of the gene in 54% of patient's. This finding suggests that not only is PKC gamma present in the colon but it may also have a role to play in enhancing colon cancer.

Having demonstrated that PKC gamma expression was expressed at high levels in the colon cancer patient tissue, we next considered the functional consequences of suppressing PKC gamma in colon cancer cells. Interestingly, when we suppressed PKC gamma in colon cancer cells there was a marked reduction in the size of spheroids developed in 3-dimensional culture. Further to this, we observed a dramatic decrease in the cells ability to form colonies in plating efficiency assays. Another key hallmark of cancer progression is an increase in the migratory capacity of cancer cells [42, 43]. Strikingly, we observed a significant reduction in the ability of cells to migrate when PKC gamma levels were reduced. This equates with previous research which demonstrates that PKC gamma phosphorylates nonmuscle myosin heavy chain II-B (NMHC-IIB) to increase cell motility of prostate cancer cells [38]. Moreover, a study examining PKC gamma in colon cancer cells revealed that PKC gamma interacts with Fascin and Rac at the edge of the cells to promote cell migration [37]. Taken together, this data suggests that PKC gamma has a tumour promoting role in colon cancer.

We next examined the effect of suppressing PKC gamma on cell adhesion and proliferation and we observed a significant increase in both. Recent work has emerged clearly demonstrating that reduced proliferation is an integral part of the migratory phenotype [44, 45]. Interestingly, the latter study reveals that hyperproliferation is important for the initiation and maintenance of primary tumours but growth inhibition is crucial for the survival of carcinoma cells and hence leads to a more metastatic phenotype. Moreover, it has been demonstrated that PKC gamma is detected in higher levels in cell lines derived from advanced or metastatic colon cancer cells [46]. Considering this, we propose that PKC gamma may drive the migratory capacity inhibiting the proliferation of colon cancer cells. However, to fully elucidate this hypothesis rescue experiments in which the phenotype is reversed would need to be conducted.

Collectively, the results in this study provide a strong argument that PKC gamma plays a key role in colon cancer and may have therapeutic benefits. However, animal model studies would need to be conducted to confirm that overexpression of the gene coding PKC gamma contributes to the metastatic potential of colon cancer. Following on from this it may be appropriate to consider drugs which target PKC gamma specifically.

There is a growing body of evidence demonstrating a diversity of roles for the different PKC isozymes [20]. Consequently, the exact role of PKCs in cancer remains elusive, highlighting the importance of revisiting exactly how we target PKCs. The dogma that PKCs act as tumour promoters led to the development of several target therapies against cancer [4–6]. However, PKC inhibitors have proved unsuccessful as anti-cancer agents in clinical trials [4, 20, 32]. Further to this, recent evidence has emerged indicating a tumour suppressor role for PKCs [7–9]. Much confusion surrounding the role of PKCs arises from contradictory immunohistochemical and biochemical studies which indicate altered expression of the PKC isozymes [21, 47, 48]. Recent commentary suggested that one of our biggest challenges in targeting PKCs is the limited data available on patient samples, and, the difficulties associated with translating animal models in the clinic [32]. Here, using human tissue we have clearly demonstrated the abundant expression of the gene coding PKC gamma in the colon tissue.

This human expression data combined with our cell based assays strongly suggests that PKC gamma plays a role in colon cancer. This study together with the emerging contradictory arguments showing both tumour suppressor and tumour promoter roles for PKCs [20] clearly highlights the need to revisit individual PKC isozymes in a tissue specific manner.

## MATERIALS AND METHODS

### Clinical samples

A cohort of colorectal tissue samples were collected, with ethical approval from the University Hospital Limerick's Ethics Committee, from 23 patients (median age 69 y; range, 45–85; male 15, female 8) undergoing surgery in University Hospital Limerick. One patient had stage 1, 14 patients had stage 2 and 8 patients had stage 3 colon cancer. Normal tissue from the 23 patients was also collected approximately 10 cm away from the tumour tissue. Specimens were immediately placed in Allprotect tissue reagent (Qiagen) and stored at −80°C.

### RNA extraction and cDNA synthesis

Frozen tissue was immersed in liquid nitrogen and ground into powder. Lysis buffer was added to tissue and the sample transferred to tubes using a 21-gauge needle. Total RNA was extracted as per Qiagen RNeasy Mini Kit instructions. RNA was quantified using a Nanodrop Spectrophotometer (Thermo Scientific) and stored at −80 degrees. RNA purity was evaluated by the ratio of absorbance at 260/280 nm and RNA quality was evaluated through visualization of the 28S:18S ribosomal RNA ratio on a 1% agarose gel. Total RNA (1 μg) was synthesised into cDNA using Vilo cDNA synthesis kit (Invitrogen) and stored at −20 degrees.

### Real-time PCR

Real-time PCR was conducted using the ABI 7900 HT instrument (Applied Biosystems) following supplier instructions. Taqman^®^ Gene Expression Assay Kits (Applied Biosystems) were used to analyse the gene expression of the PKC gamma coding gene. A panel of nine housekeeping genes were evaluated using excel Normfinder and the four most stable genes were used for normalisation of each experiment.

### Cell culture and transfection

HCT116, HT29 and LS174T colorectal carcinoma cells were purchased from ATCC (ATCC^®^ CCL-247™ with certificates of analysis) and were maintained in Dulbecco's modified Eagle's medium (DMEM) (Sigma), supplemented with 10% (v/v) fetal calf serum, 10 mM L-Glu, and 5 mg/ml penicillin/streptomycin. Cells were transiently transfected with siRNA oligonucleotide against PKC gamma and a control oligonucleotide, using the NEON transfection system^®^ (Thermo Fisher Scientific) as per manufactures instructions. After 24 h in culture, cells were treated as described in the figures. PKC gamma levels were analysed using a total PKC gamma antibody (Santa Cruz, sc-211).

### 3-Dimensional cell cultures

Individual wells of a 6 well plate were coated with Matrigel^TM^ (BD Biosciences) and placed in an incubator at 37°C for 30 min. Transfected cells were trypsinized and counted. 50,000 cells/ml were resuspended in DMEM supplemented with 2% Matrigel^TM^. Cells were placed in Matrigel^TM^ coated wells for 30 min at 37°C, after which DMEM supplemented with 2% Matrigel^TM^ was added to the cultures. Cells were maintained in culture for 6 days in an incubator at 37°C, 5% CO_2_ with fresh medium added every 2 days and cultures imaged every 24 hours (Olympus cellSens Dimension 1.12).

### Colony formation assay

Transfected cells were harvested with trypsin/EDTA, washed with DMEM and counted using a haemocytometer. 500 cells were plated per well of a 6 well plate and incubated at 37°C in 5% CO_2_ for 10 days. Following this time, the cells were fixed in 96% ethanol for 10 min and subsequently stained with 0.05% crystal violet for 20 min. The wells were washed carefully and allowed to dry. Colonies were counted and recorded and a colony was deemed to be of 50 cells or more in size.

### Scratch wound assay

Adhesive wound assay inserts (Ibidi) were used to generate wounds in confluent layers in a 6 well plate. To do this, transfected cells were trypsinized and seeded into the plate to form a monolayer. After overnight growth and attachment, the insert was carefully removed and the cells were starved of serum by adding serum free media for 4 hours, after which DMEM was added to the cells and they were imaged immediately (T0) using (Olympus cellSens Dimension 1.12). Cells were maintained in an incubator at 37°C, 5% CO_2_ for 24 hours, after which time images of the wound closure were taken as described above. The percentage wound closure was analysed using Wimasis image analysis.

### Proliferation and adherence assay

The rate of proliferation and adherence was monitored in real time using the xCELLigence E-plate system. Either 30,000 HCT116 cells transfected with siRNA oligonucleotide against PKC gamma or 30,000 HCT116 cells transfected with a control oligonucleotide were placed in each well. The impedance value of each well was automatically monitored by the xCELLigence system (xCELLigence RTCA DP, ACEA) for duration of 24 hours and expressed as a cell index value (CI). For cell growth assays cells were transfected as above and seeded at 50,000 cells, following 24 and 48 hours of growth, cells were trypsinised and counted in triplicate.

### Statistical analysis

Statistical analysis was performed using SPSS 20 Statistical Package unless stated otherwise. Significance between two groups was determined by the Mann Whitney *U* Test. For matched patient samples differences in gene expression levels was determined for each individual patient using Pair Wise Fixed Reallocation Randomisation Test^©^ as per REST^©^ software. For all statistical analysis differences were considered to be statistically significant at *p* < 0.05.

## SUPPLEMENTARY MATERIALS FIGURE


