# Supplementary material for: Expression of protein kinase C gamma promotes cell migration in colon cancer
Source: Oncotarget. 2017 Jul 1;8(42):72096–107. doi: 10.18632/oncotarget.18916 (PMC5641114; doi:10.18632/oncotarget.18916)
Supplement: Supplementary file 1 [file oncotarget-08-72096-s001.pdf]

Expression of protein kinase C gamma promotes cell migration in colon cancer

Supplementary Materials

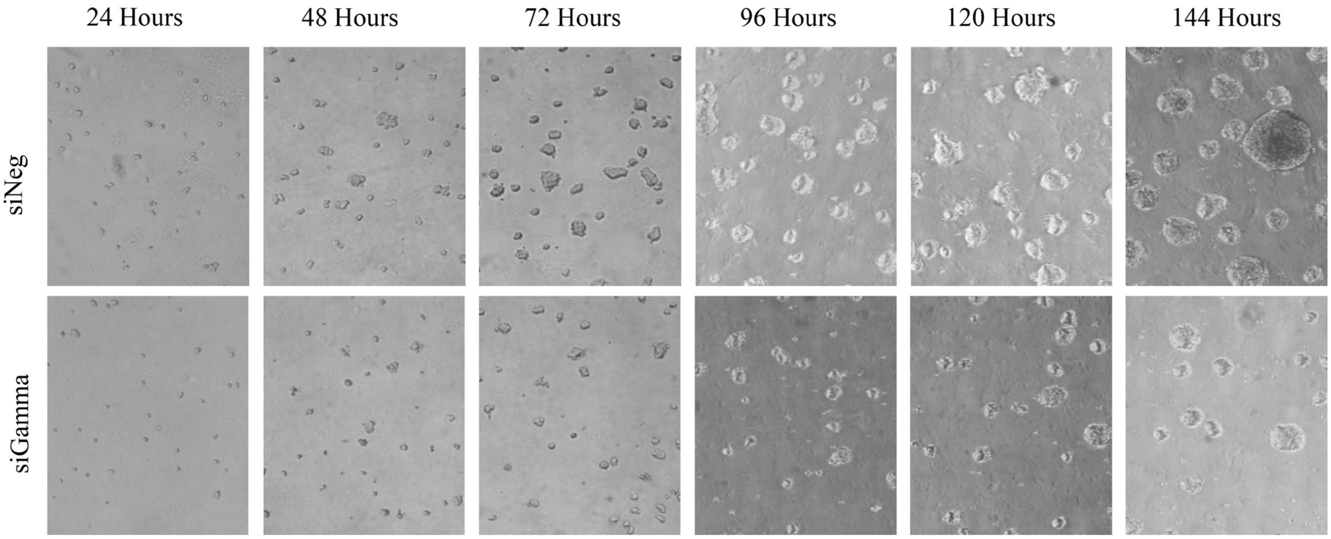

Supplementary Figure 1: 3D Spheroid Images. Images demonstrating the difference in the size of cells growing in a 3D matrix at 24 hour intervals.
